# Endogenous levels of cytokinins, indole-3-acetic acid and abscisic acid in *in vitro* grown potato: A contribution to potato hormonomics

**DOI:** 10.1038/s41598-020-60412-9

**Published:** 2020-02-26

**Authors:** Martin Raspor, Václav Motyka, Slavica Ninković, Petre I. Dobrev, Jiří Malbeck, Tatjana Ćosić, Aleksandar Cingel, Jelena Savić, Vojin Tadić, Ivana Č. Dragićević

**Affiliations:** 1 Department of Plant Physiology, Institute for Biological Research “Siniša Stanković” – National Institute of Republic of Serbia, University of Belgrade, Bulevar Despota Stefana 142, 11060 Belgrade, Serbia; 2 Laboratory of Hormonal Regulations in Plants, Institute of Experimental Botany of the Czech Academy of Sciences, Rozvojová 263, CZ-165 02 Prague 6, Czech Republic; 3 Laboratory of Mass Spectrometry, Institute of Experimental Botany of the Czech Academy of Sciences, Rozvojová 263, CZ-165 02 Prague 6, Czech Republic; 4 Mining and Metallurgy Institute, Zeleni Bulevar 35, 19219 Bor, Serbia; 5 Department of Plant Physiology, Faculty of Biology, University of Belgrade, Studentski trg 16, 11000 Belgrade, Serbia

**Keywords:** Auxin, Cytokinin

## Abstract

A number of scientific reports published to date contain data on endogenous levels of various phytohormones in potato (*Solanum tuberosum* L.) but a complete cytokinin profile of potato tissues, that would include data on all particular molecular forms of cytokinin, has still been missing. In this work, endogenous levels of all analytically detectable isoprenoid cytokinins, as well as the auxin indole-3-acetic acid (IAA), and abscisic acid (ABA) have been determined in shoots and roots of 30 day old *in vitro* grown potato (cv. Désirée). The results presented here are generally similar to other data reported for *in vitro* grown potato plants, whereas greenhouse-grown plants typically contain lower levels of ABA, possibly indicating that *in vitro* grown potato is exposed to chronic stress. Cytokinin *N*-glucosides, particularly *N*7-glucosides, are the dominant cytokinin forms in both shoots and roots of potato, whereas nucleobases, as the bioactive forms of cytokinins, comprise a low proportion of cytokinin levels in tissues of potato. Differences in phytohormone composition between shoots and roots of potato suggest specific patterns of transport and/or differences in tissue-specific metabolism of plant hormones. These results represent a contribution to understanding the hormonomics of potato, a crop species of extraordinary economic importance.

## Introduction

Plant hormones, also called phytohormones, are major factors controlling plant growth and development. They are divided into several classes based on chemical structure and biological roles. Major phytohormone classes include cytokinins, auxins, abscisic acid, gibberellins, ethylene, brassinosteroids, jasmonates, salicylic acid and strigolactones^1,2^.

Cytokinins (CKs) are a group of plant hormones with a wide spectrum of biological roles. Natural CKs share the structure of *N*^6^-substituted adenine, whereby the nature of the *N*^6^-substituent defines them as either *isoprenoid* or *aromatic*^3^. The adenine-like structure of CKs and their biosynthetic dependence on adenine metabolism suggest their close physiological connection to the processes including growth and cell division, in which the actively dividing cells produce large amounts of nucleobases and ribosides, including adenine and adenosine, for de novo synthesis of DNA.

In isoprenoid CKs, the *N*^6^-atom of adenine is substituted with an isoprenoid (C_5_) chain derived from dimethylallyl diphosphate. This CK class is the most widespread, and consists of *N*^6^-(Δ^2^-isopentenyl)adenine (iP), *cis*-zeatin (*c*Z), *trans*-zeatin (*t*Z), dihydrozeatin (DHZ), and their conjugates. *Cis*- and *trans*-zeatin isomers are *cis*- and *trans*- hydroxy derivatives of iP, respectively, whereas DHZ represents a saturated form of zeatin. Structural differences in CK side chains dictate differences in receptor binding – which determines their physiological role, but also in substrate affinity for enzymes involved in their metabolism, further causing differences in their metabolic regulation^3–5^.

Free CK bases (also called CK nucleobases: *c*Z, *t*Z, DHZ and iP) can be converted to a number of classes of molecular conjugates (the system of their abbreviations adopted and modified according to Kamínek *et al*.^6^) such as:

- ribosides (*c*ZR, *t*ZR, DHZR and iPR) – products of ribosylation of the *N*^9^-atom of the adenine ring of *c*Z, *t*Z, DHZ and iP, respectively;
- ribonucleotides (CK phosphates)- riboside-5′-monophosphate (RMP), -diphosphate (RDP) or -triphosphate (RTP) derivatives;
- *O*-glucosides and *O*-xylosides – products of glucosylation or xylosylation of the oxygen atom in the side chains of *c*Z, *t*Z, DHZ and their respective ribosides^5,7^;
- *N*-glucosides – products of *N*3-, *N*7- or *N*9-glucosylation of the adenine ring of *c*Z, *t*Z, DHZ or iP^5^.

**Figure 1:**
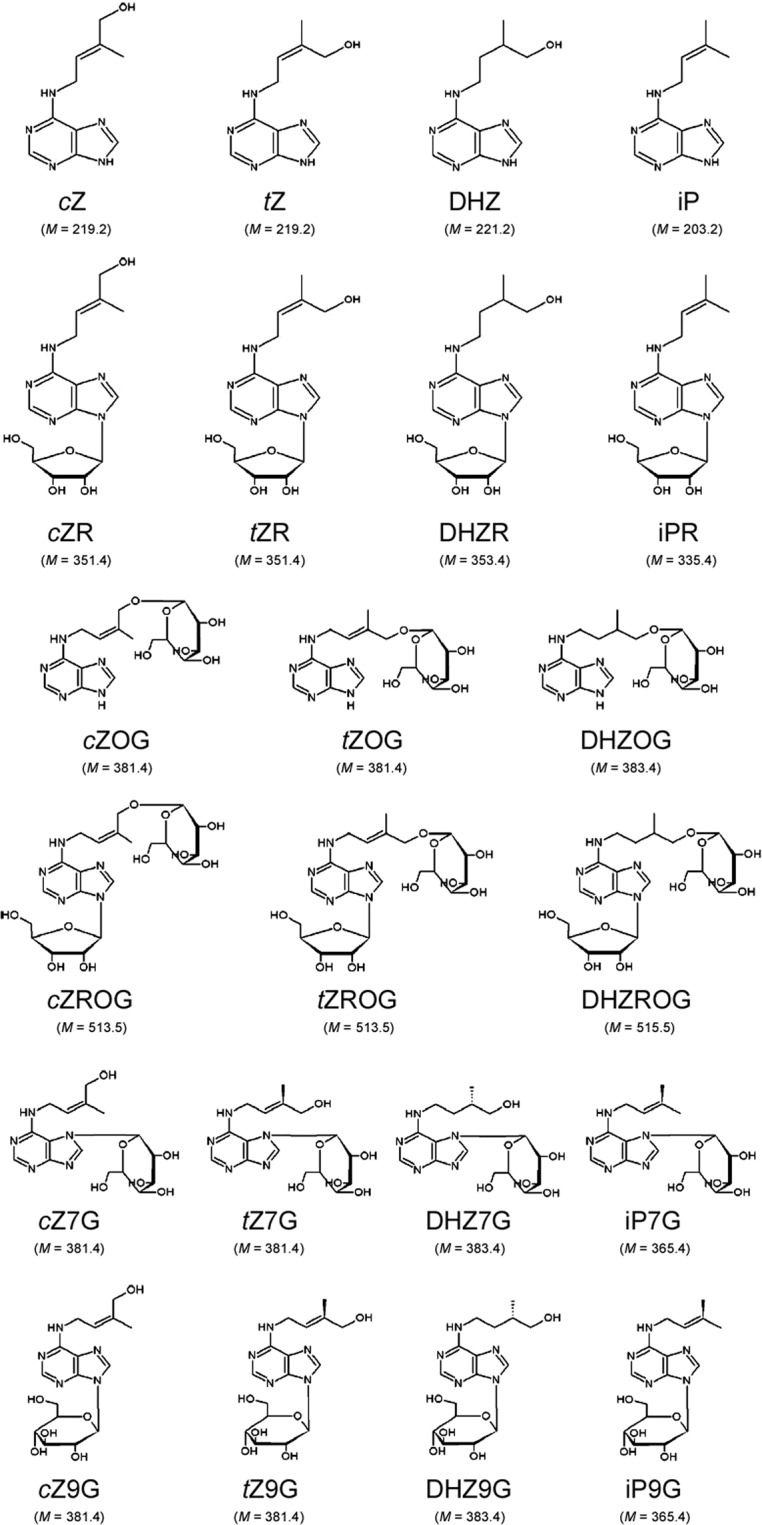
Molecular structure of isoprenoid cytokinins. Molar mass (*M* [g/mol]) of each cytokinin molecule is given below the corresponding formula. *c*Z – *cis*-zeatin; *t*Z – *trans*-zeatin; DHZ – dihydrozeatin; iP – *N*^6^-(Δ^2^-isopentenyl)adenine; *c*ZR – *cis*-zeatin 9-riboside; *t*ZR – *trans*-zeatin 9-riboside; DHZR – dihydrozeatin 9-riboside; iPR – *N*^6^-(Δ^2^-isopentenyl)adenine 9-riboside; *c*ZOG – *cis*-zeatin *O*-glucoside; *t*ZOG – *trans*-zeatin *O*-glucoside; DHZOG – dihydrozeatin *O*-glucoside; *c*ZROG – *cis*-zeatin 9-riboside *O*-glucoside; *t*ZROG – *trans*-zeatin 9-riboside *O*-glucoside; DHZROG – dihydrozeatin 9-riboside *O*-glucoside; *c*Z7G – *cis*-zeatin 7-glucoside; *t*Z7G – *trans*-zeatin 7-glucoside; DHZ7G – dihydrozeatin 7-glucoside; iP7G – *N*^6^-(Δ^2^-isopentenyl)adenine 7-glucoside; *c*Z9G – *cis*-zeatin 9-glucoside; *t*Z9G – *trans*-zeatin 9-glucoside; DHZ9G – dihydrozeatin 9-glucoside; iP9G – *N*^6^-(Δ^2^-isopentenyl)adenine 9-glucoside. The system of cytokinin abbreviations is adopted and modified according to Kamínek *et al*.^6^. Structural formulas are adapted according to Bajguz and Piotrowska^5^.

The structures and molar masses of CK nucleobases, ribosides, *O*-glucosides, *N*7- and *N*9-glucosides are shown in Fig. 1.

Biological activity of CK conjugates, primarily CK ribosides, has been debated for long, but recent work has proven that within plant membranes, CK receptors are only able to effectively bind CK nucleobases, thus making them the only genuine bioactive forms^8^. Conversions of CK nucleobases into ribosides, nucleotides, *O*-glucosides, *O*-xylosides and *N*3-glucosides are reversible processes, whereas *N*7- and *N*9-glucosylations are irreversible^5,9^. Ribosylation and *O*-glucosylation are believed to play a major role in temporary modification of CKs for purposes of transport and storage, respectively^4^. Ribosides are the main form in which CKs are transported through the plant. The current model proposes that CKs are transported through the xylem mostly in the form of *t*ZR, which, upon unloading in leaves, undergoes metabolic conversion either into the biologically active *t*Z, or into storage forms by means of *O*-glucosylation^4,10,11^. Conversely, phloem transportation occurs in the form of iPR^4,10^. On the other hand, *O*-glucosylation is thought to be the most suitable for purposes of CK storage, as a reversible but chemically stable modification, resistant to catabolic degradation by cytokinin oxidase/dehydrogenase^4,12^. *N*7- and *N*9-glucosides are also stable molecules but *N*7- and *N*9-glucosylations are chemically irreversible and do not allow further regeneration of biologically active CKs^3,5^.

Aromatic CKs, named for the aromatic substitution of the *N*^6^-atom of the adenine ring, have only recently been acknowledged as a widespread group of CKs, likely to play important roles in biological processes that might be distinct from those regulated by isoprenoid CKs^13,14^, but data on their occurence in plant tissues have remained scarce until recently, when their universal presence in plants has been suggested^7,14^.

It is believed that CKs occur in all plants. Isoprenoid CKs represent one of the most abundant phytohormone groups, having been found in almost all plants examined to date, including algae^15^ and bryophytes^16^. In vascular plants, their levels typically amount to 1–100 μg/g fresh weight (FW), being the highest in seeds and storage organs^4^. The most abundant CK of higher plants is *t*Z; it is also believed to have the most important biological role. The iP-type CKs are also highly abundant, mostly in the form of iPR. The importance of *c*Z-type CKs in plant development has been denied for long, but in the last years their involvement has been confirmed in a variety of physiological processes^7,17^.

There is growing research interest for CK physiology in potato (*Solanum tuberosum* L.) due to numerous evidence for the involvement of CKs in the commercially important process of tuberisation^18–23^. A number of reports containing measurements of phytohormones in various tissues of potato plants grown in various conditions (*in vitro*, greenhouse) have been published so far^22,24–33^. Data on amounts of particular molecular forms of isoprenoid CKs, as well as the auxin indole-3-acetic acid (IAA), and abscisic acid (ABA), measured in control plants in these studies, are summarised in Table 1. In addition, a report on endogenous levels of aromatic CKs in both *in vitro* and greenhouse-grown potato plants was published by Baroja-Fernández *et al*.^34^.

**Table 1:** Endogenous levels of particular cytokinin molecules and indole-3-acetic acid (IAA) and abscisic acid (ABA) in various tissues of potato, as reported in available literature data.

Publication	Tissue	Hormone	Endogenous amount reported	In pmol/g tissue		Cultivar or subspecies	Grown
Schmülling *et al*.^24^	shoots	IAA	962 pmol/gFW			T342 (“empty” transformation vector)	greenhouse
		ABA	112 pmol/gFW				
		*t*ZR	9.2 pmol/gFW				
		DHZR	4.1 pmol/gFW				
		iPR	2.9 pmol/gFW				
	roots	IAA	975 pmol/gFW				
		ABA	37 pmol/gFW				
		*t*ZR	3.1 pmol/gFW				
		DHZR	2.0 pmol/gFW				
		iPR	4.2 pmol/gFW				
Dermastia *et al*.^25^	whole plantlets	*t*Z	10.0 pmol/gFW			Sante	*in vitro*
		DHZ	9.5 pmol/gFW				
		iP	not detected				
		*t*ZR	164.2 pmol/gFW				
		DHZR	17.5 pmol/gFW				
		iPR	55.8 pmol/gFW				
		*t*Z9G	21.6 pmol/gFW				
		DHZ9G	35.5 pmol/gFW				
		iP9G	44.2 pmol/gFW				
Yakovleva *et al*.^26^	leaves	*t*Z	2–16 ng/gFW		9–73 pmol/gFW	Kamyk	*in vitro*
		*t*ZR	6–15 ng/gFW		17–43 pmol/gFW		
Macháčková *et al*.^27^	shoots	IAA	14.2 ng/gFW		81 pmol/gFW	Miranda	*in vitro*
		ABA	200–300 ng/gFW		750–1150 pmol/gFW		
		*t*Z	11.2 ng/gFW		51 pmol/gFW		
		iP	10.1 ng/gFW		50 pmol/gFW		
		*t*ZR	20.1 ng/gFW		57 pmol/gFW		
		iPR	12.5 ng/gFW		37 pmol/gFW		
Macháčková *et al*.^28^	shoots	IAA	200–400 ng/gFW		1100–2300 pmol/gFW	*S. tuberosum* *ssp. andigena*	greenhouse
		ABA	10–25 ng/gFW		38–95 pmol/gFW		
	leaves	IAA	200–250 ng/gFW		1100–1400 pmol/gFW		
		ABA	10–40 ng/gFW		38–150 pmol/gFW		
De Jong *et al*.^29^	leaves	ABA	300–560 ng/gDW		1100–2100 pmol/gDW	not stated	greenhouse
Muñiz García *et al*.^30^	leaves	ABA	68.2 ng/gFW		258 pmol/gFW	Spunta	*in vitro*
Kolachevskaya *et al*.^31–33^	shoots	IAA	332 pmol/gDW			Désirée	*in vitro*
		ABA	2089 pmol/gDW				
		*c*Z	104 pmol/gDW				
		*t*Z	58.5 pmol/gDW				
		iP	206 pmol/gDW				
		*c*ZR	1535 pmol/gDW				
		*t*ZR	404 pmol/gDW				
		DHZR	56.1 pmol/gDW				
		iPR	3329 pmol/gDW				

Type of tissue where endogenous phytohormones were measured, potato cultivar and growth conditions (greenhouse or *in vitro*) are disclosed for each measurement. For scientific reports where control plants or tissues were compared to plants or tissues that had undergone specific treatments, only values corresponding to control plants or tissues are provided. The system of cytokinin abbreviations is adopted and modified according to Kamínek *et al*.^6^. FW = fresh weight; DW = dry weight.

Comparison between values for endogenous levels of phytohormones in Table 1 is complicated by the use of different measurement units (pmol or ng per 1 g FW or dry weight – DW) in different studies. Conversion between pmol/gFW and ng/gFW can be carried out using molar mass of a given molecule as the conversion factor. On the other hand, it is impossible to make precise conversions between units per 1 g FW and DW, but generally, concentrations per 1 g DW are expected to have up to 10-fold higher values than corresponding concentrations per 1 g FW, as DW typically corresponds to 2-9-fold larger values of FW, depending on the water content of plant tissues^35^.

Among the publications listed in Table 1, only few include reports on endogenous levels of certain CK conjugates^25^ or *c*Z-type CKs^32,33^ in potato. The first report on the whole CK profile of potato shoots and roots (cv. Désirée, grown *in vitro*) has been published by our research group in 2012, dividing CKs into four groups based on their conjugation status and physiological function, but without breaking them down into individual molecular forms^22^.

Recent biotechnological approaches increasingly rely on manipulating the metabolism and signal transduction pathways of plant hormones, making the understanding of hormonomics a necessary step in endeavors to enhance productivity and resistance to stress of economically important plant species^36^. The present work is intended as a contribution to potato hormonomics, attempting to provide the first complete CK profile, including all analytically detectable molecular forms of isoprenoid CKs in both shoots and roots of potato (cv. Désirée) grown *in vitro*. Additionally, we present the levels of IAA and ABA and compare these data to other reports available in literature.

## Results

**Figure 2:**
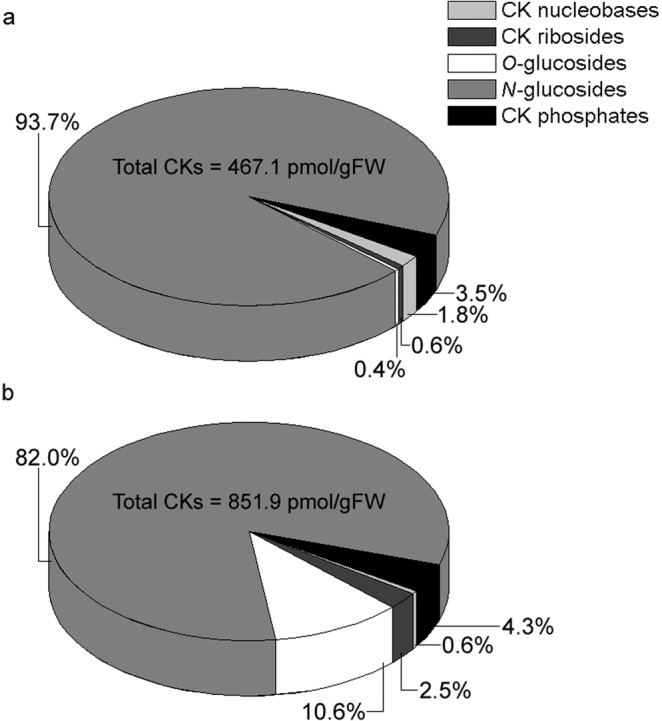
Quantitative composition of endogenous cytokinins in shoots (**a**) and roots (**b**) of 30 day old *in vitro* grown potato (*Solanum tuberosum* L. cv. Désirée) plants. The absolute amount of total cytokinins for each tissue is shown inside each of the two pie charts. Data represent means of three biological replicates.

The composition of endogenous CKs in shoots and roots of potato plants grown *in vitro* is summarised in Fig. 2. Total CK amount in the roots (851.9 ± 16.4 pmol/gFW) was almost double as abundant as in shoots (467.1 ± 5.8 pmol/gFW). The most abundant group of CKs in both shoots and roots were *N*-glucosides, accounting for 94% of total CK pool in the shoots and 82% in the roots, while bioactive CK nucleobases comprised only a small portion of total CK content in both shoots and roots of potato.

**Figure 3:**
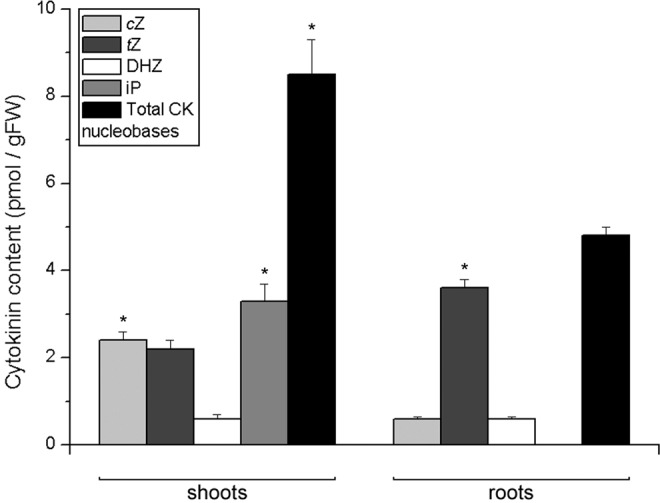
Endogenous levels of cytokinin nucleobases in shoots (left) and roots (right) of 30 day old *in vitro* grown potato (*Solanum tuberosum* L. cv. Désirée) plants. Data represent means ± standard errors (*n* = 3). Asterisks (*) are used to indicate the tissue with the higher prevalence of a CK molecule, where differences between shoot and root content are statistically significant (*P* < 0.05 according to Student’s *t*-test).

Endogenous levels of CK nucleobases in shoots and roots of potato plants are shown in Fig. 3. Their amount in shoots was significantly higher than in roots. The most abundant CK nucleobase in the shoots was iP, closely followed by *c*Z and *t*Z, while DHZ was less abundant. In the roots, the most abundant nucleobase was *t*Z; endogenous levels of *c*Z and DHZ were much lower, while iP was not detected in potato roots in our experiment.

**Figure 4:**
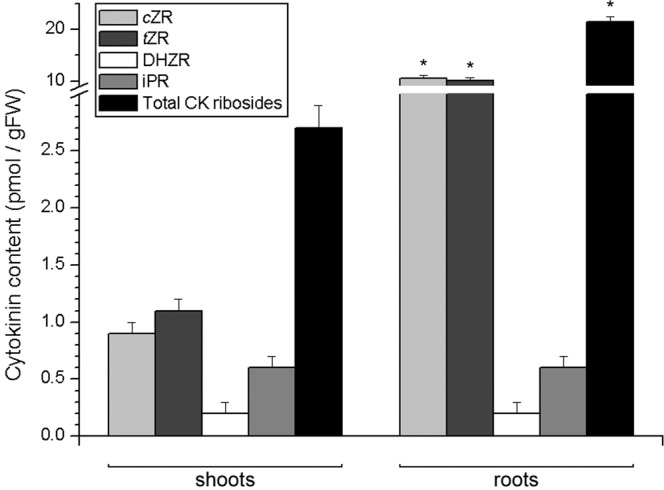
Endogenous levels of cytokinin ribosides in shoots (left) and roots (right) of 30 day old *in vitro* grown potato (*Solanum tuberosum* L. cv. Désirée) plants. Data represent means ± standard errors (*n* = 3). Asterisks (*) are used to indicate the tissue with the higher prevalence of a CK molecule, where differences between shoot and root content are statistically significant (*P* < 0.05 according to Student’s *t*-test).

To the contrary from nucleobases, CK ribosides were around 8 times more abundant in potato roots than in shoots (Fig. 4). The most abundant riboside in potato shoots was *t*ZR, followed by *c*ZR and iPR, while DHZR was present barely at the detection level. In roots, the two isoforms of zeatin ribosides, *c*ZR and *t*ZR accounted for most of the high levels of CK ribosides, while iPR was much less abundant and DHZR barely at the detection level.

**Figure 5:**
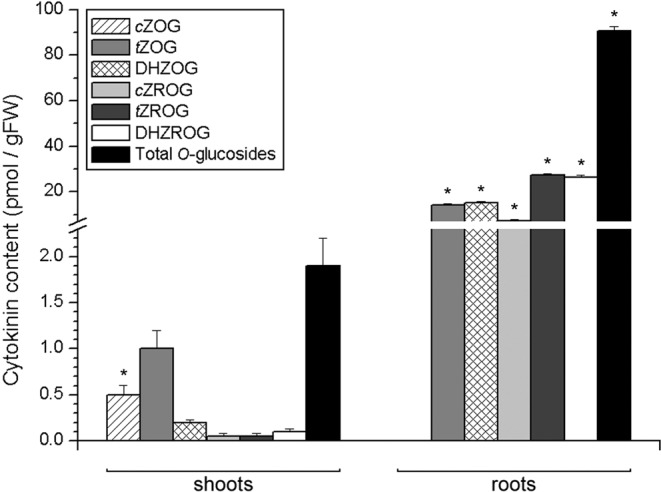
Endogenous levels of cytokinin *O*-glucosides in shoots (left) and roots (right) of 30 day old *in vitro* grown potato (*Solanum tuberosum* L. cv. Désirée) plants. Data represent means ± standard errors (*n* = 3). Asterisks (*) are used to indicate the tissue with the higher prevalence of a CK molecule, where differences between shoot and root content are statistically significant (*P* < 0.05 according to Student’s *t*-test).

Cytokinin *O*-glucosides were almost 50 times more abundant in roots than in shoots of potato (Fig. 5). In potato shoots, *t*ZOG was the most abundant *O*-glucoside, followed by *c*ZOG, while other *O*-glucosides were present at less than 0.2 pmol/gFW each. In roots, the most abundant *O*-glucosides were *t*ZROG and DHZROG, accounting for nearly one-third of the total amount of *O*-glucosides each, while DHZOG, *t*ZOG and *c*ZROG accounted for the rest, and *c*ZOG was not detected.

**Figure 6:**
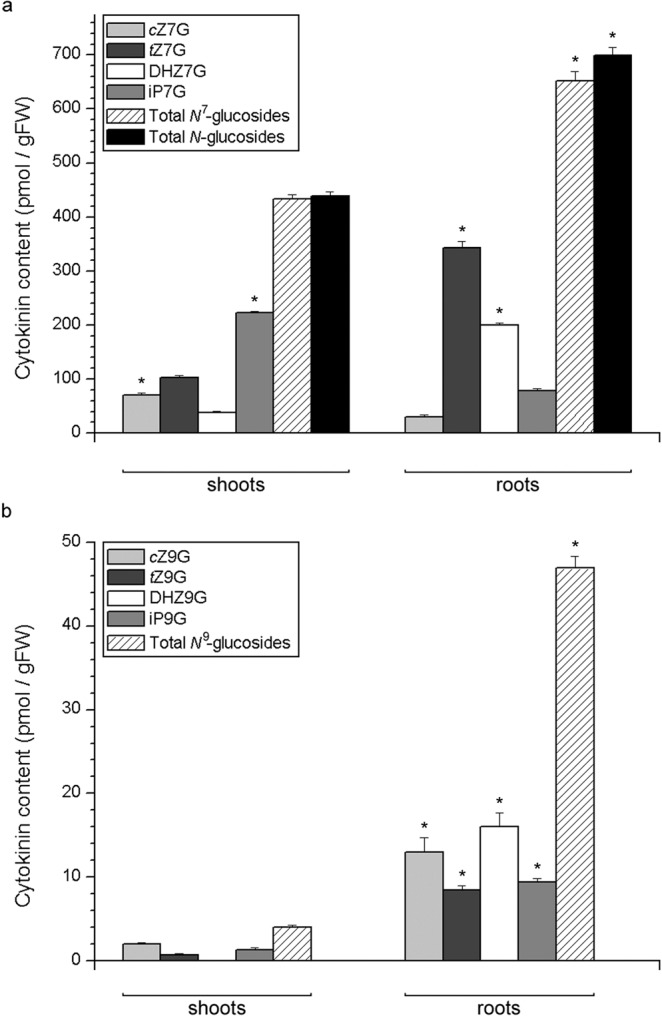
Endogenous levels of cytokinin *N*-glucosides in shoots and roots of 30 day old *in vitro* grown potato (*Solanum tuberosum* L. cv. Désirée) plants. Total *N*-glucosides (**a**) are dominantly comprised of *N*7-glucosides (**a**) while *N*9-glucosides (**b**) are present in much lower amounts. Data represent means ± standard errors (*n* = 3). Asterisks (*) are used to indicate the tissue with the higher prevalence of a CK molecule, where differences between shoot and root content are statistically significant (*P* < 0.05 according to Student’s *t*-test).

Cytokinin *N*-glucosides represented the most abundant type of CKs in potato. Endogenous levels of *N*7- and *N*9-glucosides are presented in Fig. 6a,b, respectively. Remarkably high levels of *N*7-glucosides accounted for 99.1% of total *N*-glucosides (and 92.9% of total CKs) in the shoots, and 93.3% of total *N*-glucosides (and 76.5% of total CKs) in the roots of potato (Fig. 6a). Half of the total *N*7-glucosides in the shoots was comprised by iP7G, followed by *t*Z7G, *c*Z7G and DHZ7G. In the roots, the dominant *N*7-glucoside was *t*Z7G, accounting for around half of the total *N*7-glucosides, and followed by DHZ7G, while iP7G and *c*Z7G were relatively less abundant, although still present at high levels (Fig. 6a). Of the *N*9-glucosides, *c*Z9G was the most abundant in the shoots, followed by iP9G and *t*Z9G, while DHZ9G was not detected. Differently from the shoots, DHZ9G was the most abundant *N*9-glucoside in the roots, followed by *c*Z9G, while iP9G and *t*Z9G were somewhat less abundant. All the *N*9-glucosides had significantly higher levels in roots than in shoots (Fig. 6b).

**Figure 7:**
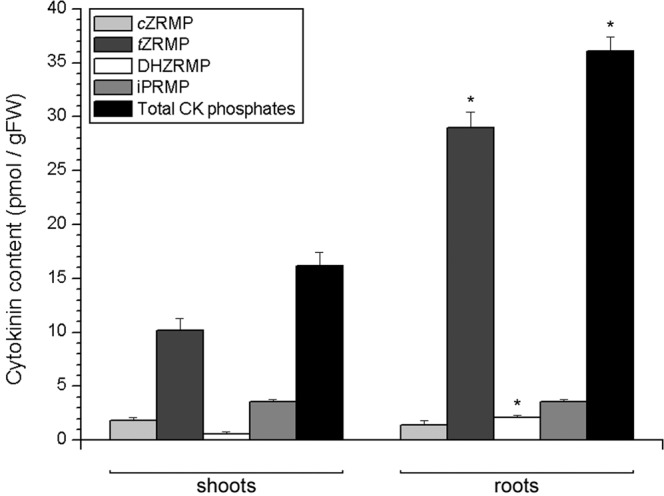
Endogenous levels of cytokinin phosphates in shoots (left) and roots (right) of 30 day old *in vitro* grown potato (*Solanum tuberosum* L. cv. Désirée) plants. Data represent means ± standard errors (*n* = 3). Asterisks (*) are used to indicate the tissue with the higher prevalence of a CK molecule, where differences between shoot and root content are statistically significant (*P* < 0.05 according to Student’s *t*-test).

Cytokinin phosphates in potato roots were more than double as abundant as in shoots (Fig. 7). The most abundant in both tissues were *t*Z-phosphates, accounting for 63% of all CK phosphates in the shoot and 80% in the root. They were followed by iP-phosphates in both tissues, while *c*Z-phosphates and DHZ-phosphates were less abundant. The levels of *t*Z-phosphates and DHZ-phosphates were significantly higher in the roots compared to shoots.

Endogenous content of IAA as the most important naturally occurring auxin, is shown in Table 2. There was no statistically significant difference between endogenous levels of IAA in shoots and roots, but the IAA/CK nucleobase ratio was significantly higher in roots (13.0) than in shoots (9.6). Table 2 also shows the endogenous levels of ABA, which were significantly higher in shoots (251.9 pmol/gFW) than in roots (124.3 pmol/gFW), while no significant difference between shoots and roots was shown for the ABA/CK nucleobase ratio.

**Table 2:** Endogenous levels of indole-3-acetic acid (IAA) and abscisic acid (ABA) in shoots and roots of 30 day old *in vitro* grown potato plants, and their ratio to the endogenous level of cytokinin nucleobases in respective tissues.

Plant hormone	Shoots	Roots
IAA (pmol/gFW)	82.0 ± 19.0	62.3 ± 11.9
IAA/CK nucleobase ratio	9.6 ± 2.2	13.0 ± 2.5*
ABA (pmol/gFW)	251.9 ± 14.9*	124.3 ± 7.8
ABA/CK nucleobase ratio	29.6 ± 1.8	25.9 ± 1.6

Asterisks (*) are used to indicate the greater of the two values, where differences between shoot and root are statistically significant (*P* < 0.05 according to Student’s *t*-test).

## Discussion

Potato (*Solanum tuberosum* L.) is the world’s fourth most important crop species, after maize, wheat and rice. Its worldwide production in 2017 amounted to 388 million tonnes grown on 19.3 million hectares (http://www.fao.org/faostat/en/#data/QC). The economic importance of potato has motivated extensive research in the area of potato biology, including the recent sequencing of the 844 Mbp-large potato genome^37^.

Particular research attention has focused on the process of tuberisation in potato, both for its economic importance, and for the suitability of potato as a model system for this physiological trait that is not widespread in the plant world. Phytohormones have been considered major players in regulating tuberisation. Gibberellins have been the first group of hormones shown to be importantly involved in tuberisation^38^, but in the recent years growing attention has been paid also to other groups of phytohormones, primarily CKs and auxins^18–23^. It has been suggested that endogenous bioactive CKs affect tuber induction, as CK-deficient potato plants overexpressing genes for cytokinin oxidase/dehydrogenase, an enzyme involved in CK catabolism, were able to undergo tuberisation before the 30^th^ day of growth in “long days” (16 h light/8 h darkness photoperiod), which never happens in wild-type plants^22,23^. The recent increase in research interest for CKs and auxins in potato and their supposed role in tuberisation has resulted in novel developments in potato physiology, most notably the identification of the three CK receptor genes in diploid Phureja potato (orthologous to the three CK receptor genes of *Arabidopsis*), and cloning and characterisation of the six receptor genes in the autotetraploid cultivar Désirée^39^. On the other hand, literature data concerning the CK hormonomics of potato have only been partial, containing little or no information about particular molecular forms such as *O*- and *N-*glucosides, and heterogenous from the point of view of the cultivars being investigated, as well as cultivation conditions.

In the present study, endogenous levels of isoprenoid CKs were determined in shoots and roots of 30 day old *in vitro* grown potato plants of the cultivar Désirée. In addition, endogenous levels of the auxin IAA have been measured for its close relation to CK physiology, and levels of ABA have been determined as an indicator of exposure to stress, for the sake of comparison to the results of other studies where potato plants were grown *in vitro* or *ex vitro*. Plants grown *in vitro* were chosen for this investigation over soil-grown plants for the sake of reproducibility of the results. Phytohormone measurements carried out on plants grown *in vitro* are expected to be more reproducible between studies, in comparison to measurements on greenhouse- or field-grown plants, where endogenous levels of various phytohormones can be subject to considerable variations depending on environmental conditions such as soil quality and composition, temperature, light regime, or air moisture.

Our results revealed that the total CK levels amounted to 467.1 pmol/gFW (173.9 ng/gFW) in potato shoots and 851.9 pmol/gFW (332.4 ng/gFW) in the roots (Fig. 2), considerably less than 1–100 μg/gFW which is considered a typical range for total CK levels in tissues of vascular plants^4^. Values that are even considerably lower than those presented here, have been reported for seedlings of basal-media grown kohlrabi (*Brassica oleracea* var. *gongylodes*)^40^ and for *in vitro* grown centaury (*Centaurium erythraea* Rafn.)^41^. Also, higher values for total endogenous CKs in the roots of potato compared to the shoots, are consistent with the observation that roots are generally more abundant in CKs, being their primary site of synthesis^42^.

Cytokinin nucleobases comprised a low proportion of total CK levels in both shoots (1.8%) and roots (0.6%) of potato, while *N*-glucosides, which are widely considered to be irreversibly inactive, accounted for 94% of the total CK pool in the shoots and 82% in the roots (Fig. 2). A similar pattern was found in CK composition of *C. erythraea*^41^, indicating that the utilisation of only a small portion of total CKs is a common phenomenon at least among asterids, if not in broader groups of plants.

Cytokinin nucleobases were more abundant in potato shoots than in roots (Fig. 3). The most abundant CK nucleobase in the shoots in our study was iP, while in the roots it was *t*Z. iP was previously shown to be the most abundant CK nucleobase in potato shoots^32,33^. It has been shown that the biological activity of iP is important for physiological processes in plant shoots^43^. On the other hand, *t*Z is important for signalling processes in the root. In maize, *t*Z, *t*ZR and *t*Z-phosphates activate the transcription of the gene *ZmRR1* to mediate the uptake of nitrogene from the soil, whereas iP-type CKs are not likely implicated in this signalling pathway^44^. There is considerable evidence for tissue-specific conversion of iP- to *t*Z-type CKs occurring predominantly in the roots of *Arabidopsis*, providing a plausible explanation for a preferential accumulation of iP in the shoot, but *t*Z in the root tissue of this species^10,45,46^. The composition of CK nucleobases in the respective tissues of potato thus suggests substantial similarities with the tissue-specific patterns of CK biosynthesis and metabolism as reported in *Arabidopsis*.

Unlike nucleobases, CK ribosides were remarkably more abundant in potato roots than in shoots (Fig. 4). The same has been observed in *C. erythraea*^41^. The most abundant CK riboside in potato shoots was *t*ZR, and it was also remarkably abundant in the roots. In previous studies^25,27^ *t*ZR was also the most abundant CK riboside in potato. This particular CK molecule is important for root-to-shoot communication in plants, being the most abundant CK transported through the xylem sap as has been documented in a number of studies^44,47,48^. The essential role of *t*Z-type CKs for long-distance signalling was confirmed in *Arabidopsis*, where loss of function of the ABCG14 transporter, responsible for CK root-to-shoot transport, caused a decrease in levels of shoot *t*Z- and DHZ- type CKs, but not iP-types^49,50^.

The almost 50-fold difference between potato roots and shoots in endogenous levels of *O*-glucosides (Fig. 5) suggests that roots are the primary CK storage site in potato. It has been suggested that *O*-glucosylation, as well as *N*-glucosylation and possibly *O*-xylosylation, may be activated as a protective mechanism against CK degradation in potato plants that overexpress the genes for cytokinin oxidase/dehydrogenase^21–23^, thus underlining the biological importance of *O*-glucosides for maintaining hormonal homeostasis in potato.

*N*-glucosides were the most dominant CK forms, accounting for the bulk of the total CK pool in both shoots and roots of potato (Figs. 2 and 6). This large prevalence of *N*-glucosides was mostly due to *N*7-glucosides, which made up for 99.1% of total *N*-glucosides in potato shoots and 93.3% in the roots (Fig. 6a), while *N*9-glucosides were much less abundant (Fig. 6b). Interestingly, *N*-glucosides represented also the dominant CK type in *C. erythraea*, but to the contrary from potato, the bulk of centaury’s *N*-glucosides was comprised by *N*9-glucosides, whereas those glucosylated at the *N*^7^-position made up for barely more than 10% of total *N*-glucosides in both shoots and roots of centaury^41^. Apparently, although both species accumulate large amounts of CK *N*-glucosides (which is therefore likely to be a common feature in plants), the preference for accumulating *N*7- versus *N*9-glucosides is probably specific to particular taxa. In *Arabidopsis*, the enzymes UGT76C1 and UGT76C2, which are responsible for CK *N*-glucosylation, both prefer *N*7- to *N*9-glucosylation, accordingly with the relative amounts of *N*7- and *N*9-glucosides in various tissues of *Arabidopsis*^4^. If similar investigations are carried out to determine the CK profiles of other plant species, preferential patterns of accumulation of various CK types could provide us with interesting new insights regarding phylogenetic differences in CK metabolism.

Cytokinin phosphates accounted for around 4% of total CKs in both shoots and roots of potato (Fig. 7). The most abundant type of CK phosphates in both shoots and roots of potato were *t*Z phosphates. Although iPRMP was present in both shoots and roots at 3.6 pmol/gFW, *t*ZRMP was much more abundant in roots than in shoots, therefore rendering the *t*ZRMP:iPRMP ratio higher in roots than in shoots. In *Arabidopsis*, biosynthesis of *t*Z-type CKs occurs through the hydroxylation of iP mono-, di- and triphosphates into respective *t*Z phosphates, by means of the enzymes of the CYP735A family. Conversion of iP phosphates into *t*Z phosphates was more intense in roots, than in leaves and shoots of *Arabidopsis*^45^. The *t*ZRMP:iPRMP ratio found in shoots and roots of potato thus indicates similarities with the tissue-specific patterns of the biosynthesis of *t*Z-type CKs as observed in *Arabidopsis*.

Taken together, differences in CK composition between shoots and roots of potato suggest specific patterns of CK transport, and/or differences in tissue-specific metabolism of these hormones. Certain patterns might not only be tissue-specific, but also remarkably specific for a particular molecular form. For instance, *c*ZOG was the only *O*-glucoside that is completely missing in the roots, whereas all other *O*-glucosides were present well over the detection threshold (Fig. 5). At the same time, *c*ZOG was the second most abundant *O*-glucoside in the shoots, accounting for ca. a quarter of all *O*-glucosides. Whether *c*ZOG might be metabolised in the roots or transported elsewhere, this phenomenon appears to be highly specific and to apply only to *c*ZOG, but not, for instance, *t*ZOG or *c*ZROG. Similarly, DHZ9G was the most abundant *N*9-glucoside in the roots, while being completely absent in shoots (Fig. 6b). It has already been suggested that metabolic pathways of *cis*- and *trans*-zeatin type CKs might be at least partly separated from each other thanks to fine-tuned substrate specificity of enzymes involved in their metabolism, e.g., in glucosylation^51^; as most of these enzymes are activated in a tissue-specific^12^ or development-related^47^ fashion, it is likely that their substrate specificity reflects on the CK composition of respective plant tissues. Possible tissue-specific mechanisms of metabolism and transport that might underlie specific differences between shoots and roots on the level of individual molecular forms of CKs, deserve special attention and require sophisticated research approaches specifically targeting each type of CK molecule.

Our results for endogenous IAA content (82.0 pmol/gFW in shoots and 62.3 pmol/gFW in roots) were similar to others reported for *in vitro* grown potato^27,31,33^ but considerably lower than those found in greenhouse-grown plants^24^. It has been reported that the IAA content of *in vitro* grown shoots of the potato cultivar Miranda amounted to 81 pmol/gFW^27^. Also, more recent data^31,33^ for the level of IAA in *in vitro* grown shoots of the cultivar Désirée can be considered more or less corresponding to the result reported for the same cultivar in our study, having in mind that DW content values are supposed to be several-fold higher than FW values.

IAA is the auxin whose endogenous content is most commonly reported in plant physiology research, and its physiological effects and metabolic regulation are considered largely interconnected with the physiology of CKs^5^. Auxins and CKs are known to regulate each other’s endogenous levels, forming a feedback loop^52–55^. The ratio between endogenous levels of IAA and bioactive CKs is considered more relevant to a number of physiological processes in plants, than the individual levels of each of these hormones alone^56^. The IAA/CK nucleobase ratio in this work was lower in shoots (9.6) than in roots (13.0) of potato (Table 2). The opposite relation has been observed between IAA/CK nucleobase ratios in shoots and roots of centaury, which might be relevant to the capacity of this species to undergo spontaneous de novo shoot organogenesis (DNSO) from roots on hormone-free media^41^, which has not been observed in potato. A high endogenous IAA/bioactive CK levels ratio was considered relevant to the ability of kohlrabi seedlings to successfully undergo DNSO on auxin-free media, contrary to hypocotyl explants which had a significantly lower IAA/bioactive CK ratio^40^. On the other hand, Kufri Sutlej, a potato cultivar with naturally high endogenous auxin levels, was able to produce only callus in standard regeneration protocols and required the addition of an anti-auxin to the regeneration media in order to regenerate shoots^57^.

Most previous studies on *in vitro* grown potato plants reported higher endogenous levels of bioactive ABA compared to results obtained for greenhouse-grown plants (Table 1), but all of them lacked data on the content of ABA metabolites and conjugates, which can be determined using LC-MS/MS as the quantification method, rather than HPLC^58^. The physiological balance between biosynthesis and catabolism of ABA is highly dynamic, making the measurements of its metabolites and conjugates useful to complement the information on the time profile of ABA levels, in settings where the fluctuations of ABA in time are relevant^59,60^ – for instance, data on endogenous levels of ABA and its metabolites in defoliated and decapitated potato shoot segments were recently published in a study on the circadian dynamics of phototropic bending in potato^61^.

However, in this study we were mainly interested in determining the bioactive ABA in order to compare to previous reports. The endogenous levels of ABA in potato shoots in this work were similar to those in the leaves of *in vitro* grown plants of the cultivar Spunta^30^ and in the shoots of the cultivar Désirée^33^. Lower endogenous levels of ABA were reported in shoots of greenhouse-grown potato plants^24,28^. There is considerable evidence that the endogenous levels of bioactive ABA are elevated in plants exposed to environmental stress^62,63^. Plants grown *in vitro* have already been widely considered to be exposed to chronic multiple stress. It has been shown that *in vitro* grown potato plants are exposed to lower light intensity and altered composition of light spectrum^64^, greater relative humidity^65^, poor or no aeration of the culture vessels resulting in accumulation of ethylene in the proximity of cultured plants^66^, and altered balance between heterotrophic and autotrophic carbon assimilation^67^. Constant exposure to multiple stress might thus contribute to increased levels of ABA in *in vitro* grown potato.

In conclusion, we determined the endogenous levels of CKs, as well as IAA and ABA, in shoots and roots of 30 day old *in vitro* grown potato (cv. Désirée) plants. Although endogenous phytohormone levels of potato plants, grown either *in vitro* or *ex vitro*, have been reported in a number of publications to date, our report is the first where all analytically detectable forms of isoprenoid CKs, including molecular conjugates and *c*Z-type CKs, are presented individually. Our results are generally similar to other reports on *in vitro* grown potato, whereas greenhouse-grown potato plants typically have lower ABA content. Differences in phytohormone composition between shoots and roots of potato suggest specific patterns of transport and/or differences in tissue-specific metabolism of plant hormones. This report represents an important step in elucidating the hormonomics of potato, a crop species which is being extensively studied because of its great agronomic importance in the 21^st^ century.

## Methods

### Plant material

Virus-free potato tubers (*Solanum tuberosum* L. cv. Désirée) obtained from the PKB Agroeconomic Institute (Belgrade, Serbia), were used to establish shoot cultures from sprouts, as previously described^68^. Tissue cultures were propagated *in vitro* every four weeks from single-node stem cuttings as described in our previous work^22^. Cultivation media, consisting of MS basal salts^69^, were solidified with 0.7% agar and supplemeted with 3% sucrose, 0.1% inositol, and vitamins^70^. The cultivation chamber was set at 25 ± 2 °C, 16 h/8 h light/dark photoperiod with white fluorescent lamps (“Philips TL–D 58 W/54-765”, 58 W, 6200 K, 50 μmol m^−2^ s^−1^, Philips, Amsterdam, The Netherlands). Shoots and roots were isolated from 30 day old plantlets from *in vitro* culture, frozen in liquid nitrogen and subjected to phytohormone extraction.

### Phytohormone extraction and quantification

Plant material (1 gFW of potato shoot and root tissue each) was homogenised in liquid nitrogen with a mortar and pestle and extracted in a mixture of methanol, formic acid and water (15:1:4, v/v/v). Purification was carried out using the dual-mode solid-phase extraction method^71^. Concentration of CK phosphates (nucleotides) was determined as corresponding ribosides following dephosphorylation by alkaline phosphatase. Detection and quantification were carried out on a HPLC/MS system LCQ (Finnigan, San José, CA, USA) operated in the positive-ion full-scan MS/MS mode using a multilevel calibration graph with [^2^H]-labelled CKs as internal standards. The list of used [^2^H]-labelled standards is given in Supplementary Table S1. Detection limits varied between 0.05 and 0.1 pmol/sample for various CKs. Endogenous levels of free (non-hydrolysed) IAA and ABA were determined by two-dimensional HPLC (first dimension: TSP, Riviera Beach, FL, USA; second dimension: Perkin Elmer, Wellesley, MA, USA) as previously described^72^. Quantification of IAA was carried out with a fluorescence detector LC 240, while ABA was quantified on the basis of ultraviolet detection with a diode array detector 235C (both manufactured by Perkin Elmer).

### Statistical analysis

Endogenous phytohormone levels were measured in three biological replicates for both shoot and root. Each measurement was repeated twice with similar results. The results are presented as means of biological replicates ± SE based on statistical analysis performed with Statgraphics Plus Version 2.1 (Statpoint Technologies Inc., Warrenton, VA, USA) with statistical differences between the means determined by Student’s *t*-test. Graphs were designed with Origin 6.1 (OriginLab Corporation, Northampton, MA, USA).

## Supplementary information

Supplementary information
