# Supplementary material for: Endogenous levels of cytokinins, indole-3-acetic acid and abscisic acid in in vitro grown potato: A contribution to potato hormonomics
Source: Sci Rep. 2020 Feb 26;10:3437. doi: 10.1038/s41598-020-60412-9 (PMC7044434; doi:10.1038/s41598-020-60412-9)
Supplement: Supplementary file 1 — Supplementary information [file 41598_2020_60412_MOESM1_ESM.pdf]

Endogenous levels of cytokinins, indole-3-acetic acid and abscisic acid in in vitro grown potato: A contribution to potato hormonomics

Martin Raspor, Václav Motyka, Slavica Ninković, Petre I. Dobrev, Jiří Malbeck, Tatjana Ćosić, Aleksandar Cingel, Jelena Savić, Vojin Tadić, Ivana Č. Dragićević

Supplementary Information

Supplementary Table S1. [^{2}H]-labelled compounds used as internal standards for cytokinin quantification. For the determination of ribonucleotides, deuterated ribonucleotides (*t*ZRMP, DHZRMP and iPRMP) were added as internal standards prior to sample purification and dephosphorylation. *c*Z – *cis*-zeatin; *t*Z – *trans*-zeatin; DHZ – dihydrozeatin; iP – N^6-(Δ^2-isopentenyl)adenine; *c*ZR – *cis*-zeatin 9-riboside; *t*ZR – *trans*-zeatin 9-riboside; DHZR – dihydrozeatin 9-riboside; iPR – N^6-(Δ^2-isopentenyl)adenine 9-riboside; *c*ZOG – *cis*-zeatin *O*-glucoside; *t*ZOG – *trans*-zeatin *O*-glucoside; DHZOG – dihydrozeatin *O*-glucoside; *c*ZROG – *cis*-zeatin 9-riboside *O*-glucoside; *t*ZROG – *trans*-zeatin 9-riboside *O*-glucoside; DHZROG – dihydrozeatin 9-riboside *O*-glucoside; *c*Z7G – *cis*-zeatin 7-glucoside; *t*Z7G – *trans*-zeatin 7-glucoside; DHZ7G – dihydrozeatin 7-glucoside; iP7G – N^6-(Δ^2-isopentenyl)adenine 7-glucoside; *c*Z9G – *cis*-zeatin 9-glucoside; *t*Z9G – *trans*-zeatin 9-glucoside; DHZ9G – dihydrozeatin 9-glucoside; iP9G – N^6-(Δ^2-isopentenyl)adenine 9-glucoside. The system of cytokinin abbreviations is adopted and modified according to Kamínek et al. (2000)[6].

Deuterated compounds	Used as internal standard for
*t*Z7G-D_5	*t*Z7G, *c*Z7G
*t*Z9G-D_5	*t*Z9G, DHZ7G(1,2)*
*t*ZOG-D_5	*t*ZOG, *c*ZOG
DHZ9G-D_3	DHZ9G, *c*Z9G
DHZOG-D_7	DHZOG
*t*ZROG-D_5	*t*ZROG, *c*ZROG
*t*Z-D_5	*t*Z
DHZ-D_3	DHZ, *c*Z
*t*ZR-D_5	*t*ZR, DHZROG
DHZR-D_3	DHZR, *c*ZR
iP7G-D_6	iP7G
iP9G-D_6	iP9G
iPR-D_6	iPR
iP-D_6	iP

*DHZ7G represents a mixture of two optical isomers separated by HPLC.
